# Supplementary material for: Validity of the cold pressor test and pain sensitivity questionnaire via online self-administration
Source: PLoS One. 2020 Apr 16;15(4):e0231697. doi: 10.1371/journal.pone.0231697 (PMC7162430; doi:10.1371/journal.pone.0231697)

At-Home Cold Pressor Test Instructions and Interface

A brief introduction to the activity provided an overview of the activity and offered a $10 electronic gift card as compensation for attempting to complete the cold pressor test at home (S1 Figure 1). The initial questions assessed safety-related exclusion criteria (conditions exacerbated by cold or stress, current pregnancy, open cuts or sores on the non-dominant hand, after which eligible participants were asked to provide consent (see S1 Figures 2).

S1 Figure 1. Activity introduction.

Pain Sensitivity Study - Cold Pressor Test

Survey

The Cold Pressor Test is a test that has been widely used to measure pain sensitivity and tolerance. In this activity, you will be asked to submerge your hand in ice water for as long as you can tolerate.

This research activity is part of a collaboration with Grünenthal, a pharmaceutical company dedicated to finding innovative medicines to treat pain.

In order to complete this activity, you will need the following:

- A bucket or kitchen sink
- Enough ice to fill the bucket or your sink about an eighth of the way full
- Enough room temperature water to fill the bucket or sink, with the ice, three quarters of the way full

You will also need to be able to **access and use your computer (or other device) while your hand is immersed** in the bucket or sink of ice water.

Estimated time to complete (including set-up): 20-30 minutes

Compensation: You will receive a $10 eGift Card for your participation.

You can always return to this task if you currently do not have the required items or time to complete it.

S1 Figure 2. Full consent form as viewed by participants.

Consent Document for the Cold Pressor Test (CPT)

This document is an addition to our general research consent document. It provides information specific to this particular research activity. For a more complete explanation of the 23andMe research program, refer to our general research consent document.

What is this research about?

23andMe is conducting research on pain. As part of this research, we are studying how pain is perceived and tolerated by the body. To achieve this goal, we are asking people to do an activity called the cold pressor test (CPT). This is a well-known and standard research activity in pain studies. The CPT subjects people to a mildly painful experience (submerging a hand in ice water) and monitors the development of their pain response. The characteristics of the pain the CPT causes are very similar to those found in individuals suffering from chronic pain, which makes this research activity a straightforward way to look for factors that might affect the management or treatment of physical, psychological, and/or chronic pain.

Who can participate in this activity?

You must be 18 years old to do this research activity. You must also be in good health. You **MAY NOT** do this activity if you have any of the following:

- High blood pressure
- Heart disease
- Dysrhythmia
- Any other cardiovascular disorder
- History of Raynaud's phenomenon
- History of fainting or seizures
- History of frostbite
- An open cut, sore or bone fracture on or near your non-dominant hand (the one you do not usually write with)
- Any neurological disorder
- Are pregnant or think you might be pregnant

If you have one of these conditions, this activity could make your condition worse or cause you harm. You **MAY NOT** take part in this activity if you have any of these conditions.

What am I agreeing to if I consent?

If you consent, we will ask you to place your hand in a container filled with ice water. You will be asked to keep your hand in the water as long as you can. We will ask you questions about the activity and any pain you feel.

You can remove your hand from the ice water at any time. Your hand will not be in the water for more than 3 minutes.

You will receive a $10 gift card for your participation.

What are the additional benefits or risks I should be aware of?

Additional Benefits

There may be no direct benefit to you for participating in this study. However, there may be some indirect benefits, including the following:

- Data from this activity could help scientists better understand pain as well as a variety of medical conditions. Using this data, scientists could develop new or improved treatments, medications, or therapies.
- While doing this activity, you may experience altruistic feelings. These may make you feel good about yourself or about helping others.

Additional Risks

- It is likely that you will feel numbness, tingling, burning, pain or other unpleasant sensations in the hand you place in the ice water. For some people, this feeling could persist several minutes after completing the activity.
- You may feel light-headed or dizzy.
- If you spill or drip water onto an electronic device (such as your computer) while doing this activity, you could damage the electronic device.

The following risks are very unlikely to happen, but you should be aware of them:

- If you use wet electronic equipment, you could receive an electric shock or be electrocuted.
- It is possible that you could faint, which could result in a fall or other injury.

What will you do with my data?

23andMe may share your data from this activity with our qualified research partners. Before sharing your data, we will strip it of any personally identifying information (such as your name or contact information). We will also combine it with data from other people who complete this activity to mask your individual responses. Your individual information will never be shared with a third party without your explicit permission. For more details, see the general research consent document.

23andMe protects the privacy of your data using a range of physical, technical, and administrative measures. These safeguards are in accordance with current technological and industry standards. For more information, please see our privacy statement.

Do I have to do this activity?

No. Participation in this research activity is completely voluntary. Your decision will have no effect on your access to your Genetic Information or the 23andMe® Personal Genome Service.

You may stop doing this research activity at any time, for any reason.

Whom do I contact if I have questions?

If you have ***any questions or concerns about this research or need help completing the research activity***, email us at pain-study@23andme.com

If you ***suffer a research-related injury***, or if you ***have a question about subjects' rights***, please contact the Human Protections Administrator at hpa@23andme.com.

If you have ***any questions or concerns about research that you do not wish to discuss with 23andMe***, contact the following independent, impartial research review board:

- E&I Review Services
- Email: subject@eandireview.com
- Phone: 1-800-472-3241

Please choose one of the following options:

○ Yes, I'd like to participate in this research. I am at least 18 years old, I do not have any of the health conditions listed above, I have read this document, and I CONSENT to continue on to this activity.

○ No, I do not want to continue on to this activity.

Participants were then asked two additional questions as possible covariates or quality control items: hand dominance and use of pain medication on the day of the activity.

S1 Figure 3. Question about hand dominance.

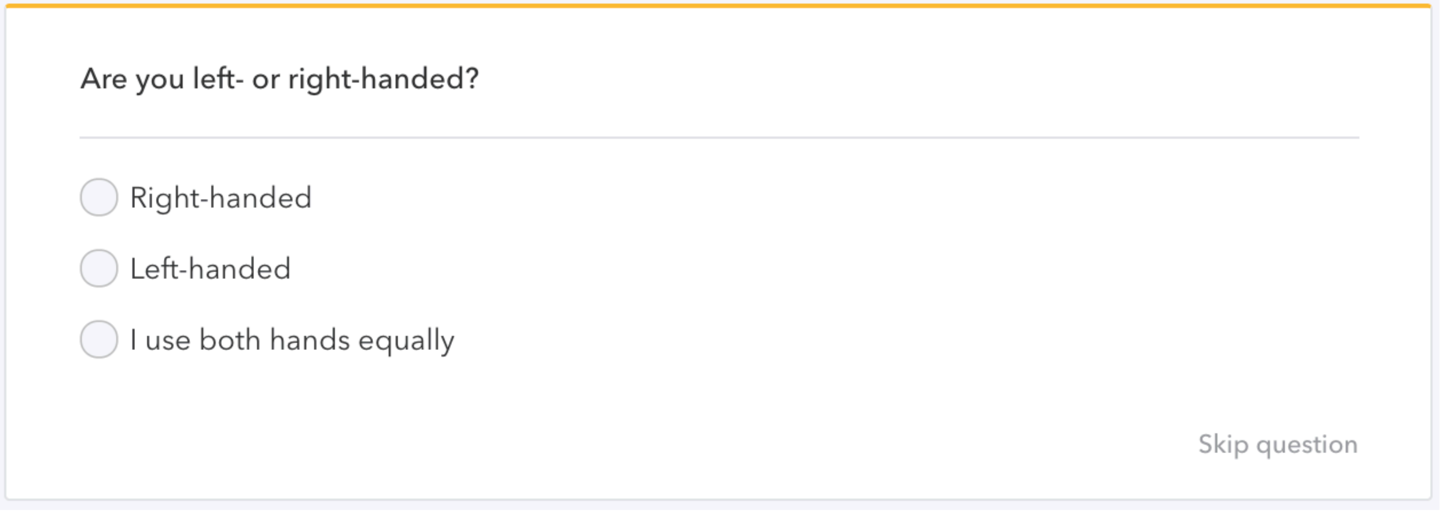

Are you left- or right-handed?

- Right-handed
- Left-handed
- I use both hands equally

Skip question

S1 Figure 4. Question about use of pain medication on the day of the activity.

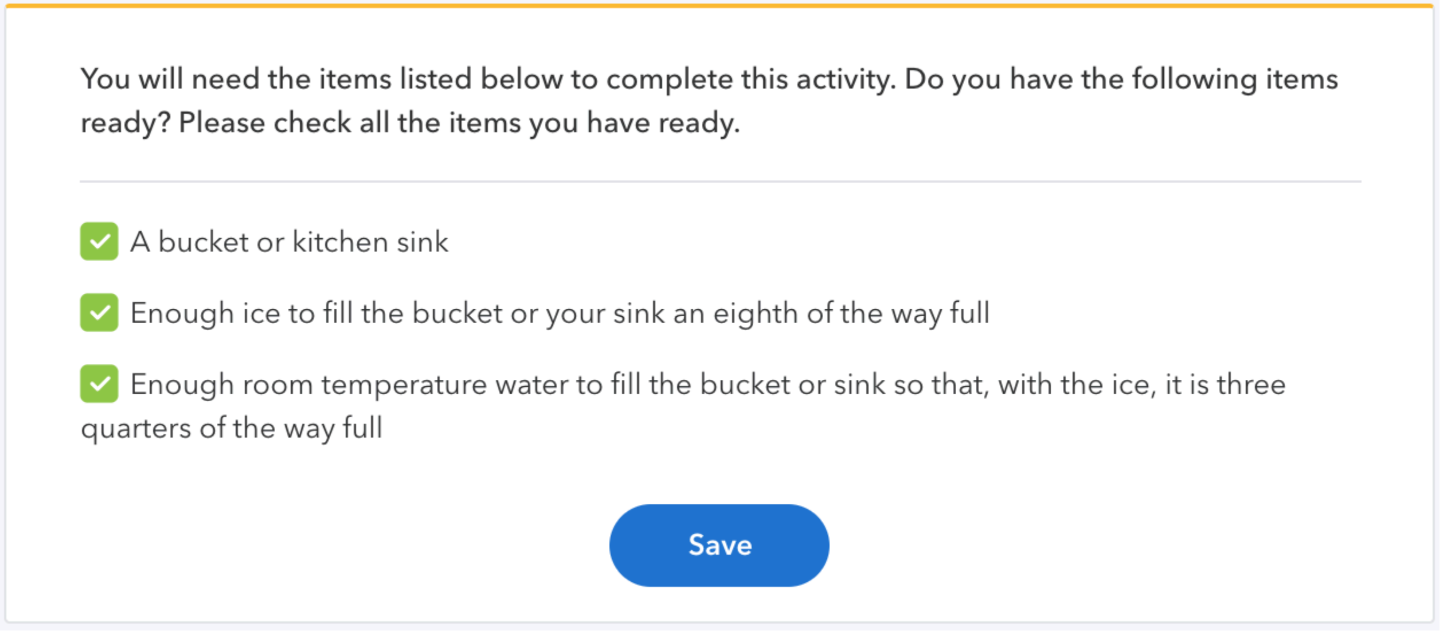

You will need the items listed below to complete this activity. Do you have the following items ready? Please check all the items you have ready.

- A bucket or kitchen sink
- Enough ice to fill the bucket or your sink an eighth of the way full
- Enough room temperature water to fill the bucket or sink so that, with the ice, it is three quarters of the way full

Save

Participants were reminded notified about the items that they would need to continue the activity, followed by instructions.

S1 Figure 5. Items required to continue the activity.

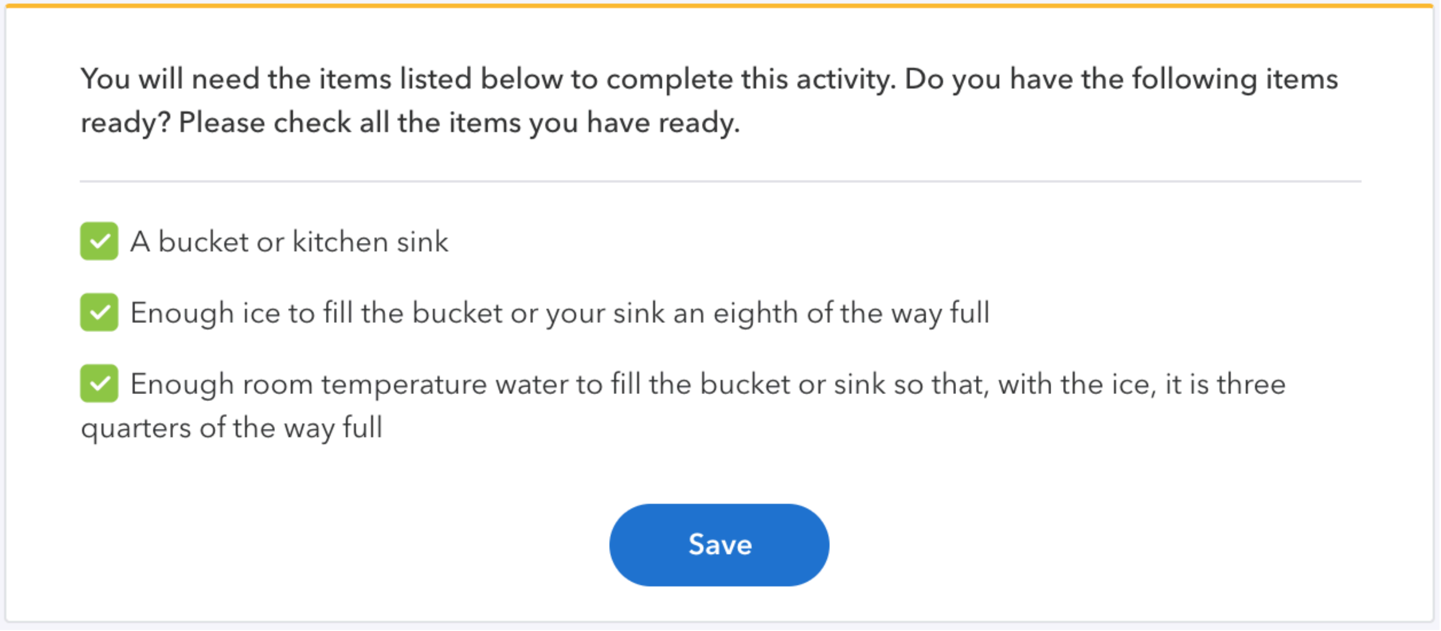

S1 Figure 6. Instructions for the activity (c.f. article Figure 1.)

Preparation:

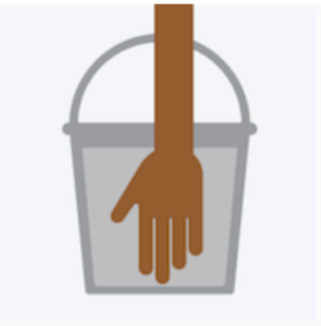

1. Check that you are **able to immerse your non-dominant hand** (the hand you do not usually write with) to just above the wrist in the bucket or sink.

   Make sure you are **able to use your computer** (or other device) with your free hand without getting water on it while doing so.

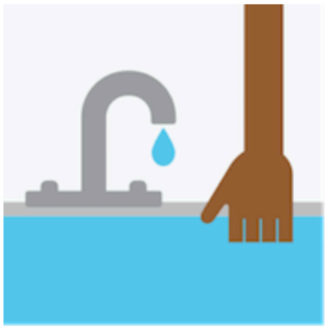

2. Check that the water you will be using is close to room temperature.

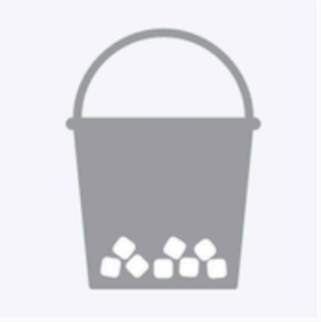

3. Fill your bucket or sink **an eighth** of the way full with ice.

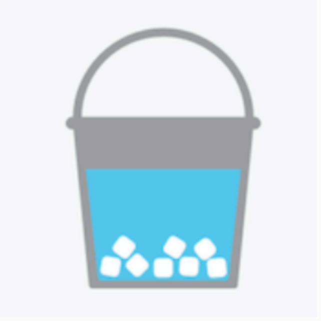

4. Add room temperature water so that, with the ice, the bucket or sink is **three quarters** of the way full.

I'm ready

We then asked that participants practice using the timer interface before starting the activity.

S1 Figure 7. Practice timer – Step 1.

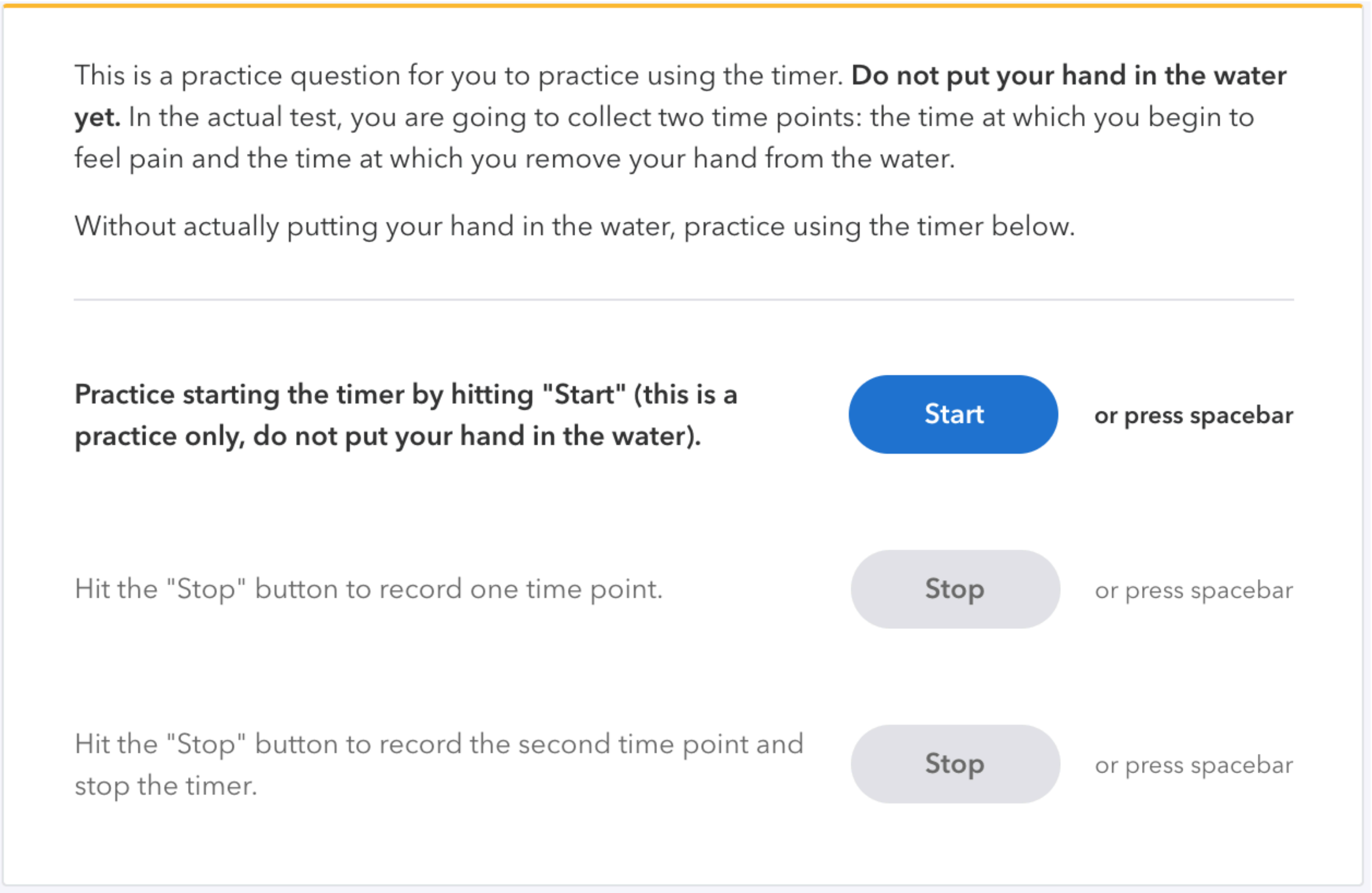

S2 Figure 8. Practice timer – Step 2.

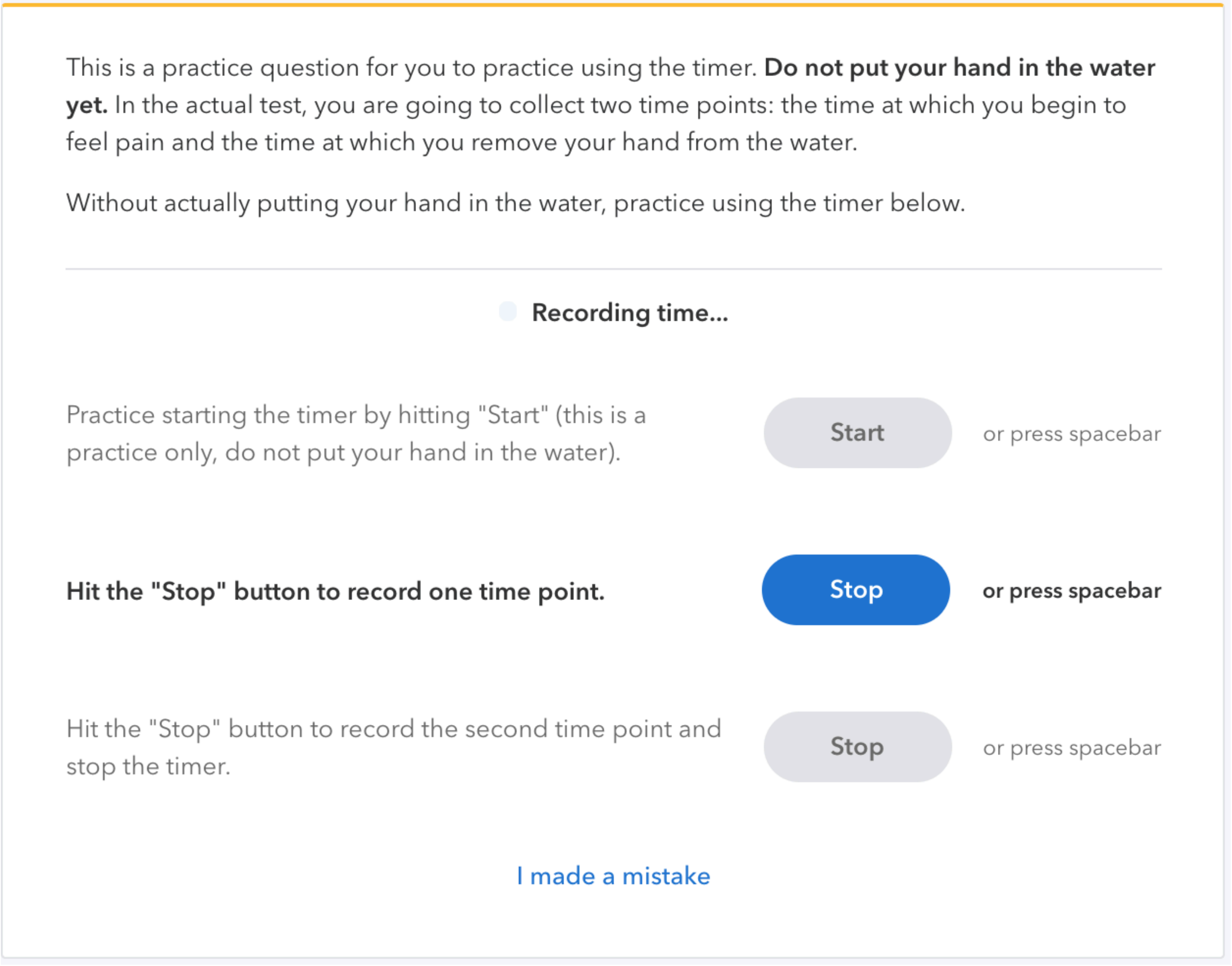

S1 Figure 9. Practice timer – Step 3.

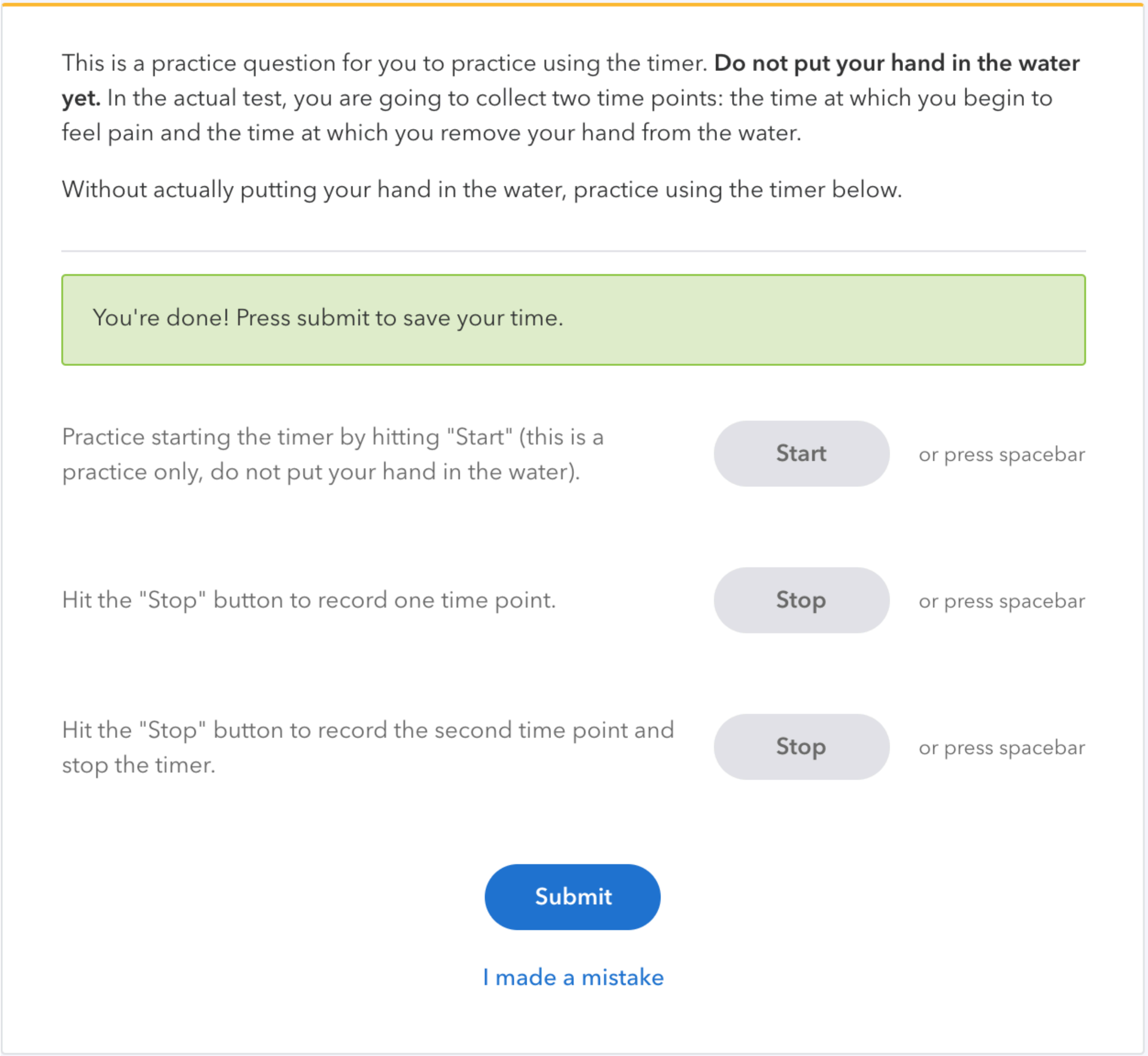

Then participants we given some additional instructions prior to start the activity itself, then started the activity.

S1 Figure 10. Pre-test instructions.

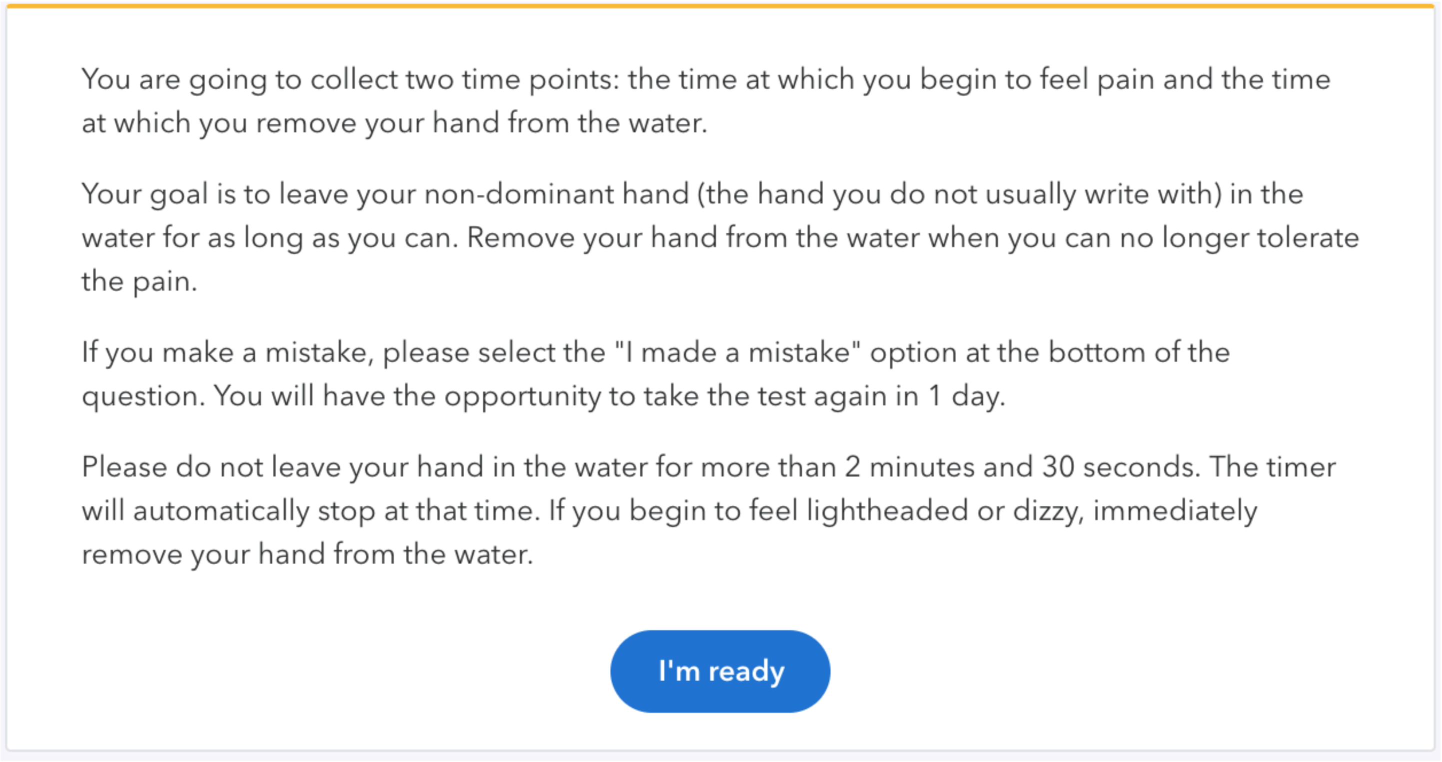

S1 Figure 11. Timer for study data collection – Step 1.

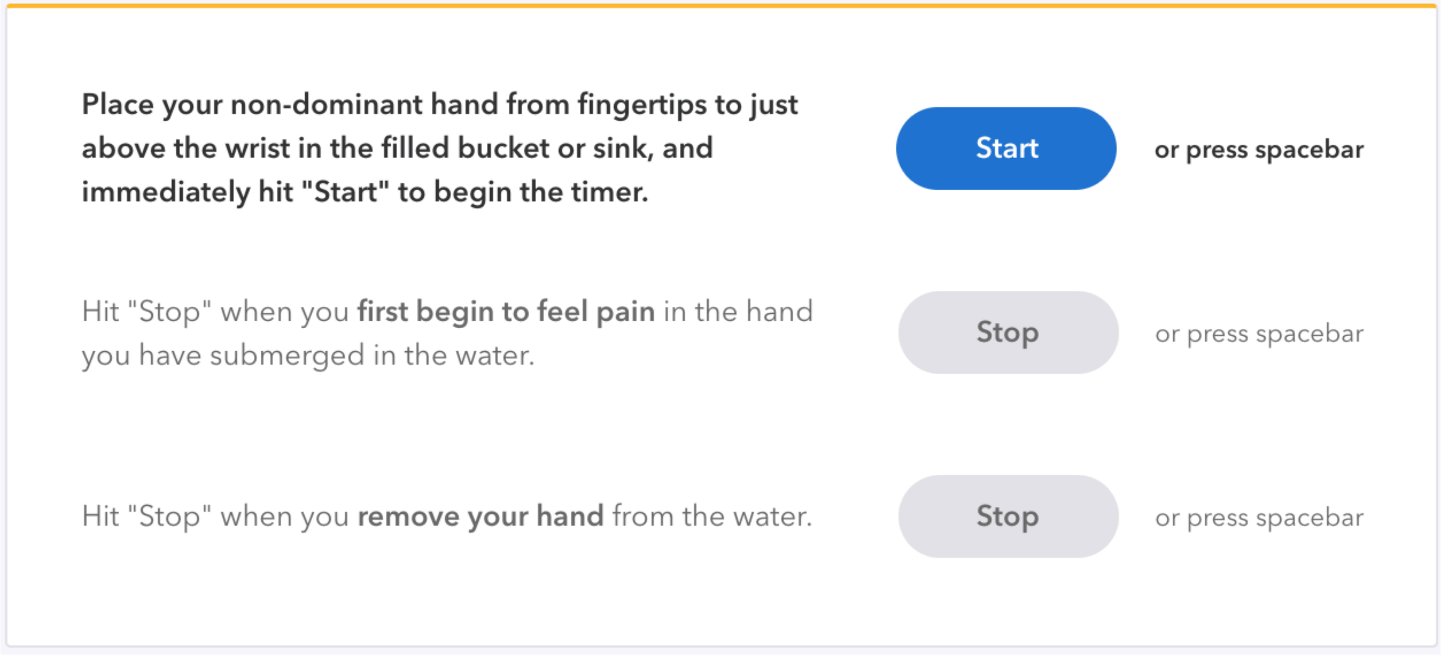

S1 Figure 12. Timer for study data collection – Step 2.

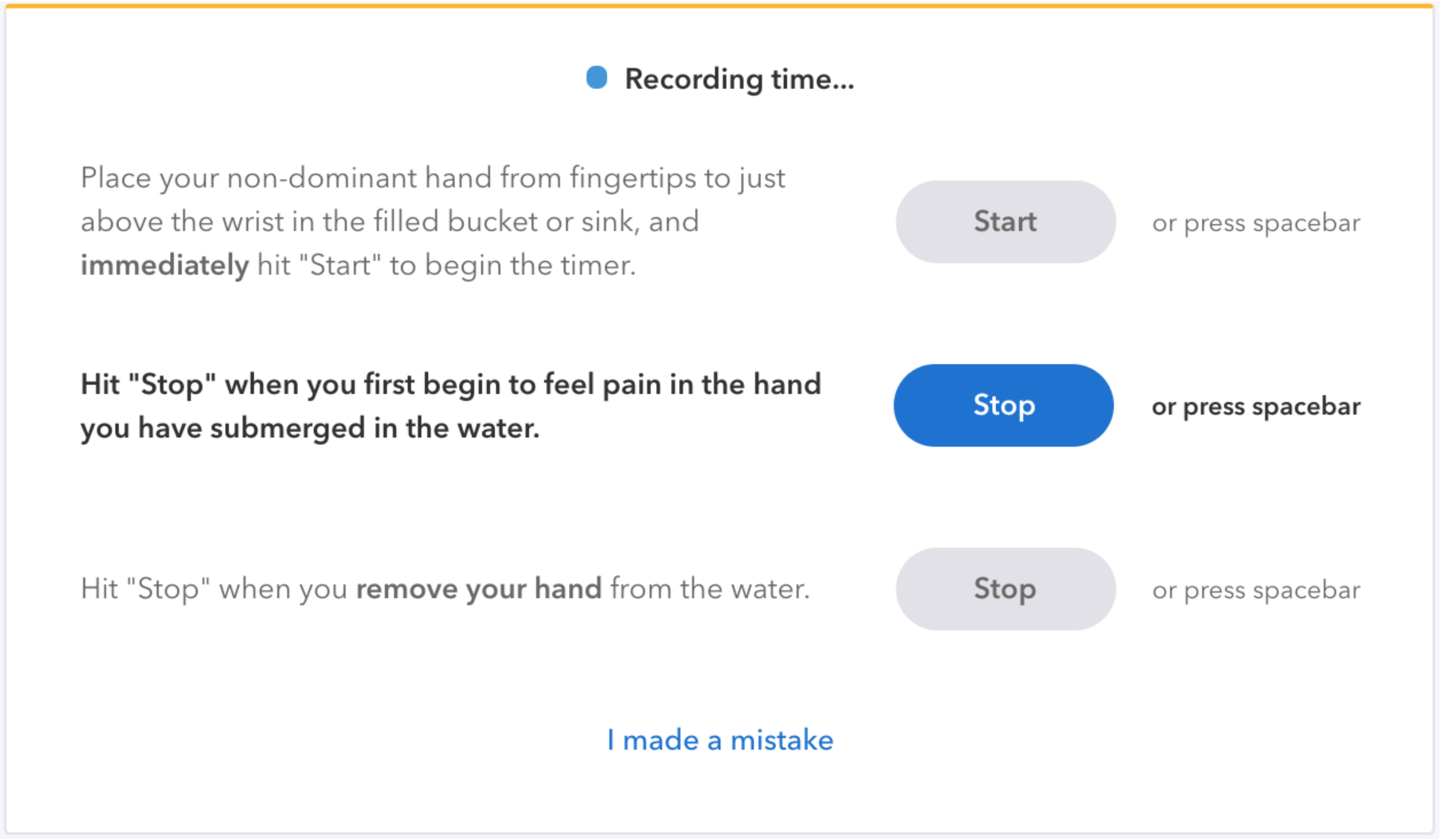

S1 Figure 13. Timer for study data collection – Step 3.

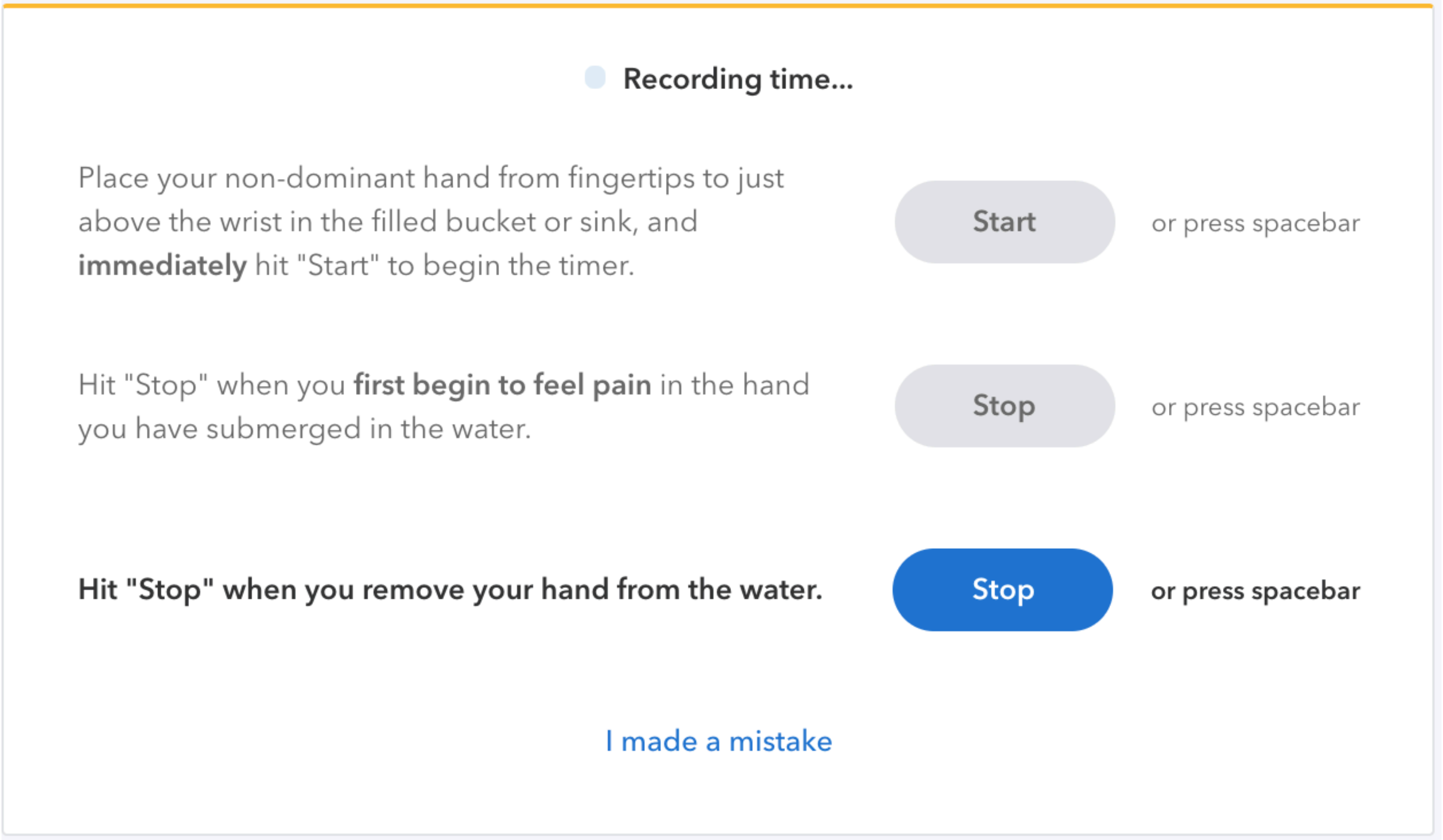

S1 Figure 14. Timer for study data collection – Step 4.

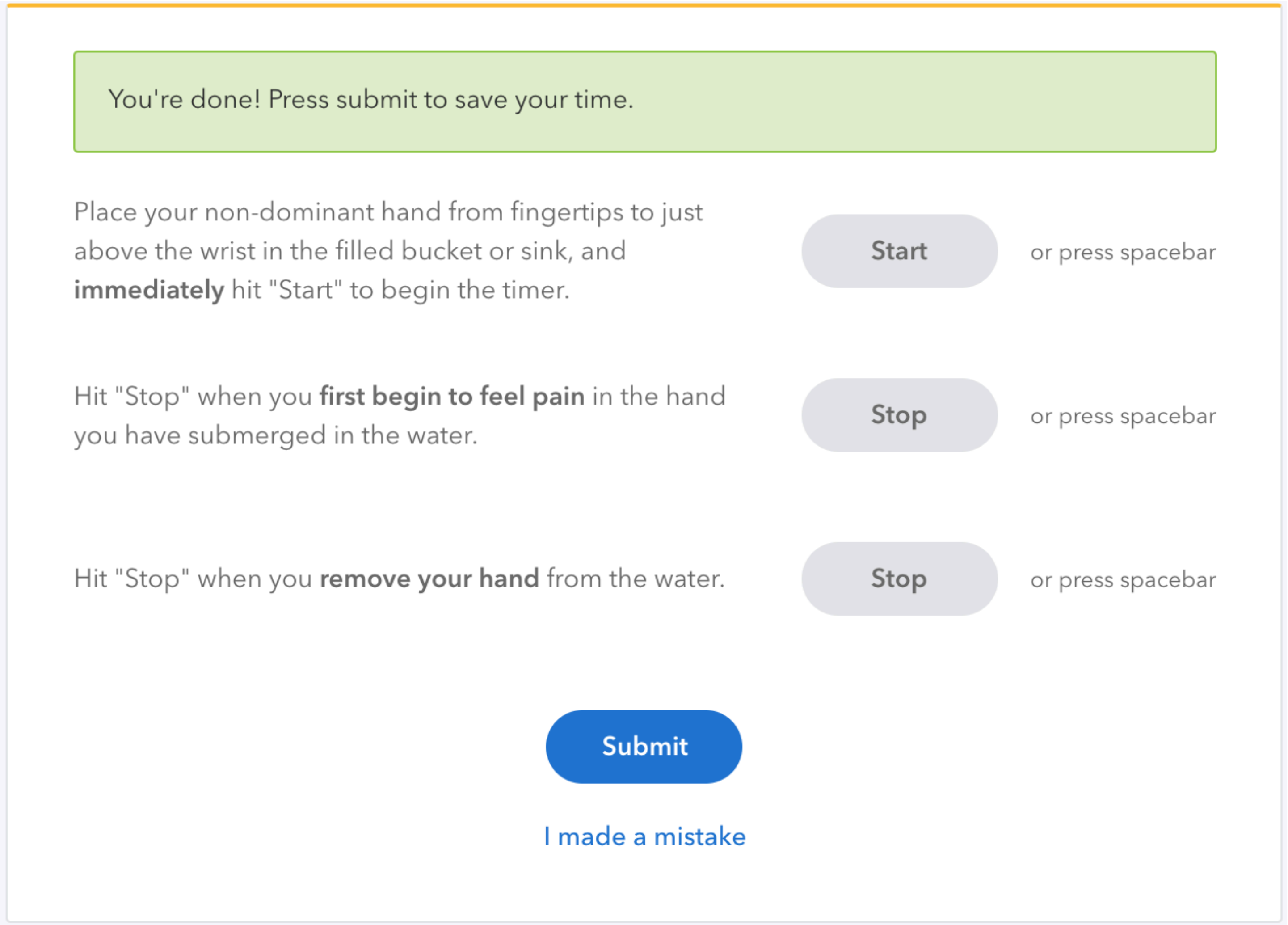

Participants who reported a mistake were asked to characterize their mistakes (e.g. not starting the time at the right time or a distraction that caused removal of the hand for a reason other than pain). Participants who did not report a mistake were asked to rate their pain at the point they removed their hands.

S1 Figure 15. Visual analog scale maximum pain rating.

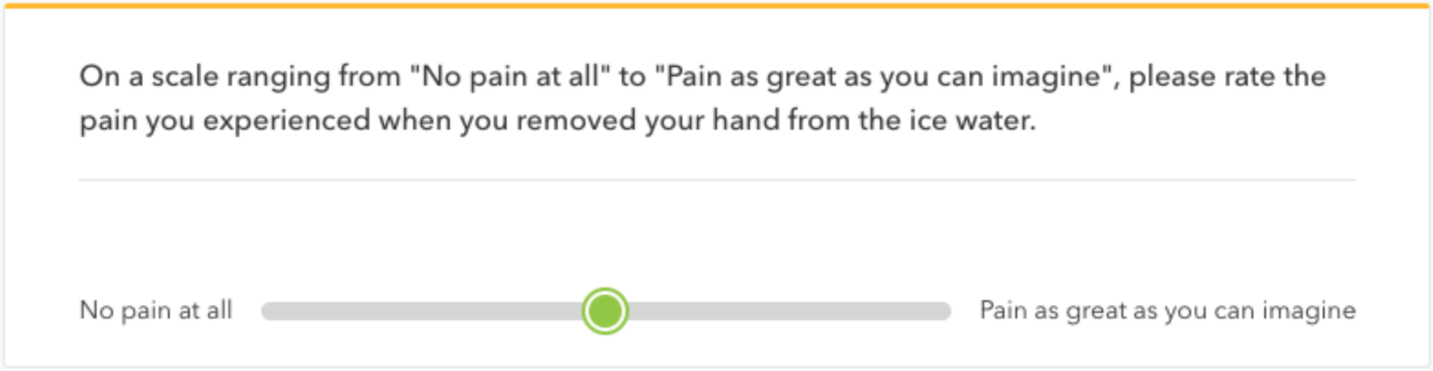

Supplement: S1 Fig — (PDF) [file pone.0231697.s001.pdf]
